# Investigations of sequencing data and sample type on HLA class Ia typing with different computational tools

**DOI:** 10.1093/bib/bbaa143

**Published:** 2020-07-14

**Authors:** Jian Yi, Longyun Chen, Yajie Xiao, Zhikun Zhao, Xiaofan Su

**Affiliations:** 1 Yucebio Cancer Translational Research Institute and Chief Medical Officer for Yucebio Technology Co; 2 Cancer Translational Research Institute, YuceBio Technology Co., Ltd., Shenzhen, China

**Keywords:** HLA-Ia genotyping, OptiType, NGS, Clinical genomics

## Abstract

Human leukocyte antigen (HLA) can encode the human major histocompatibility complex (MHC) proteins and play a key role in adaptive and innate immunity. Emerging clinical evidences suggest that the presentation of tumor neoantigens and neoantigen-specific T cell response associated with MHC class I molecules are of key importance to activate the adaptive immune systemin cancer immunotherapy. Therefore, accurate HLA typing is very essential for the clinical application of immunotherapy. In this study, we conducted performance evaluations of 4 widely used HLA typing tools (OptiType, Phlat, Polysolver and seq2hla) for predicting HLA class Ia genes from WES and RNA-seq data of 28 cancer patients. HLA genotyping data using PCR-SBT method was firstly obtained as the golden standard and was subsequently compared with HLA typing data by using NGS techniques. For both WES data and RNA-seq data, OptiType showed the highest accuracy for HLA-Ia typing than the other 3 programs at 2-digit and 4-digit resolution. Additionally, HLA typing accuracy from WES data was higher than from RNA-seq data (99.11% for WES data versus 96.42% for RNA-seq data). The accuracy of HLA-Ia typing by OptiType can reach 100% with the average depth of HLA gene regions >20x. Besides, the accuracy of 2-digit and 4-digit HLA-Ia typing based on control samples was higher than tumor tissues. In conclusion, OptiType by using WES data from control samples with the high average depth (>20x) of HLA gene regions can present a probably superior performance for HLA-Ia typing, enabling its application in cancer immunotherapy.

## Introduction

The human leukocyte antigen (HLA) complex, which is located within a 3.6-Mbp stretch on the short arm of chromosome 6 (6q21.31), can encode the human major histocompatibility complex (MHC) proteins and play a key role in adaptive and innate immunity [1, 2]. It can be divided into two categories: HLA-I (HLA-Ia, Ib) genes encode MHC-I proteins presenting in all nucleated cells and can bind to intracellular antigens for cell destruction through CD8+ cytotoxic T cells [3];HLA-II (HLA-DP, −DQ,-DR) genes encode MHC-II proteins existing in the antigen-presenting cells and can recognize intracellular and extracellular antigens for antibody production via CD4+ helper T cells. As is known, the HLA system includes the most polymorphic genes with marked differences in allele frequency between and within ethnic groups. The nomenclature of HLA allele at full resolution, like HLA-A33:03:01:02 L, orderly consists of allele groups (HLA-A33, 2-digit resolution), specific alleles (HLA-A33:03, 4-digit resolution), exon variants(HLA-A*33:03:01, 6-digit resolution), intronic variants (HLA-A*33:03:01:02, 8-digit resolution) and a tag to flag the allele expression (L, a low expression; N, a null expression) [4, 5].

In recent years, cancer immunotherapy has made significant breakthroughs and becomes one of the most widely used treatment in pan-cancer patients [6]. Emerging clinical evidence suggests that the presentation of tumor-specific intracellular antigens (neoantigens) and neoantigen-specific T cell responses associated with MHC class I molecules are of key importance to activate the adaptive immune system in cancer immunotherapy [6]. Remarkably, downregulation of HLA genes due to loss of HLA haplotype loss, downward transcriptional expressions of specific antigen presentation machinery genes and variations in tumor microenvironments, may reduce the ability to present neoantigens and promote immune evasion in many cancer types [7]. Besides, it has also demonstrated that HLA-Ia genotypes such as HLA-B44 and HLA-B62 and loss of heterozygosity (LOH) status have been shown associated with the patient prognosis and immunotherapy efficacy [8]. Moreover, a previous study found that HLA somatic mutations were detected in 8.1% and 3.3% of colorectal cancer and melanoma patients respectively, which might affect prediction accuracy [9]. Therefore, accurate HLA typing is very essential for the clinical application of immunotherapy.

HLA alleles can be typed by serological phenotype analysis or by DNA molecular analysis. Although HLA typing by serology can give a rough result in a short time, it cannot identify rare or new HLA alleles [10]. Currently, polymerase chain reaction (PCR) is the most widely used HLA typing method [11], and it can be further divided into polymerase chain reaction sequence-specific primer (PCR-SSP), polymerase chain reaction sequence-specific oligonucleotide probes (PCR-SSO) and polymerase chain reaction sequenced based typing (PCR-SBT). Among them, PCR-SBT with the highest typing accuracy is the only technique that can directly detect the nucleotide sequence of each allele. In this study, we used the SBT method based on single-molecule real-time sequencing (SMRT) technology as the gold standard.

Taking the advantages of next-generation sequencing (NGS) technologies, NGS-based HLA typing methods can detect heterozygous alleles and polymorphisms outside of traditionally amplified PCR regions, which may result in higher resolution compared to PCR-based methods [12]. However, these methods still have a lot of challenges, such as the polymorphic characteristics of HLA sequences, the limitations of NGS detection length and the high accuracy requirements for clinical application.

To overcome these challenges, those methods with alignment- or assembly-computational programs based on bioinformatic algorithms have arisen to be a potential solution. Alignment-based automatic methods compare sequencing reads with reference HLA sequences from WGS, WES or RNA-seq and forecast true alleles by using probabilistic models.

Seq2hla [13] was the first alignment method specifically for HLA typing from RNA-seq data, based on Python and R programming language. This method mapped reads to HLA nucleotide sequences from international IPD-IMGT/HLA [14] database and extracting sequences of exons 1, 2, 3 and 4 of HLA class I and II genes, by using Shannon’s entropy and binary logarithm formulation to describe HLA genotypes. Thereafter, Phlat [15] and OptiType [5] by using Python programming language were developed for DNA or RNA data. Phlat can predict HLA class I and II type from WES or RNA-seq data and was built on an alignment-filter-coverage program. The whole human genome database together with HLA alleles genomic sequences were used as the mapping reference for Phlat, and the Bayesian likelihood model was employed to predict HLA alleles [16]. OptiType based on integer linear programming was validated for WGS, WES and RNA-seq data at a full resolution for HLA class I genes. Reads were mapped against a carefully constructed reference database by extracting exons 2 and 3 sequences from each known HLA-I allele [17]. The most recent alignment method Polysolver [9] assumed Bayesian classification approach for HLA genotyping from WES data, by mapping to the reference database generated from all known HLA-I alleles in the IPD-IMGT/HLA [18]. Limited to only HLA-Ia typing, Bauer et al. showed superior performances of OptiType with 4-digit accuracy at 99% for 373 RNA-seq data from lymphoblastoid cell lines and 4-digit accuracy at 98% for 992 WES data from a phase 3 clinical study [19].

Here, we conducted performance evaluations of 4 widely used HLA typing tools (OptiType, Phlat, Polysolver and seq2hla) for predicting HLA class Ia genes from WES and RNA-seq data of 28 cancer patients. HLA genotyping data using PCR-SBT method based on SMRT technology was firstly obtained as the golden standard and was subsequently compared with HLA typing data by using NGS techniques. Besides, the impacts of depth and sample type on the tools’ accuracy were also investigated in this study. After *in silico* simulation, we finally identified the probably optimal method for HLA Ia typing.

## Results

### HLA typing performance

To evaluate the prediction performance of the 4 computational tools, raw data of WES and RNA-seq in fastq format were initially prepared for analysis. OptiType, Phlat and Polysolver were used to execute HLA typing from WES data, while OptiType, Phlat and seq2hla were utilized for RNA-seq data.

**Figure 1:**
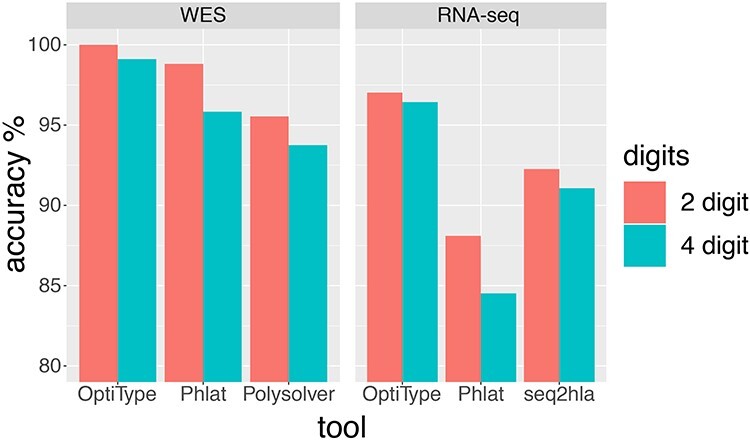
Accuracy of four computational tools on WES and RNA-seq data at 2-digit and 4-digit resolution.

As shown in Figure 1, the accuracy of OptiType, Polysolver and Phlat for 4-digit HLA typing of WES data was 99.11%, 95.83% and 93.75%, respectively. Accuracy of OptiType, Phlat and seq2hla from RNA-seq data at 4-digit resolution was 96.42%, 84.52% and 91.07%, separately. Among the other 3 tools, OptiType had the highest accuracy of HLA-Ia typing for both WES and RNA-seq data. These results suggested that OptiType might be the potential optimal choice for HLA-Ia typing, and the WES data was more accurate for HLA-Ia typing than RNA-seq data. The typing results and validation results of all WES and RNA-seq data were presented in Supplement Table S2 & S3. After removing these two samples with very low HLA gene expression (Supplementary Table S4), the accuracy of OptiType for 4-digit HLA typing from RNA-seq data was 98.72% (Table 1), indicating that low expressions of HLA genes might reduce the accuracy of HLA typing.

**Table 1:** Accuracy table of RNA-seq data for HLA-Ia genes

Data set	Tools	2-digit accuracy	4-digit accuracy
Total group (n = 28)	OptiType	97.0%	96.4%
	Phlat	88.1%	84.5%
	seq2hla	92.3%	91.1%
Selected group^*^ (n = 26)	OptiType	99.4%	98.7%
	Phlat	94.9%	91.0%
	seq2hla	98.1%	96.8%

^*^Two samples with very low HLA gene expression were removed from the total group (Supplementary Table S4).

### Impact of depth

**Figure 2:**
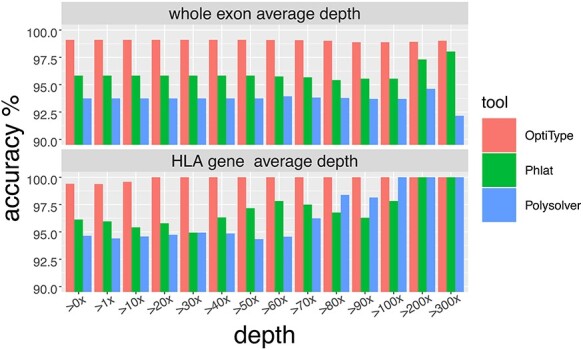
The HLA-Ia 4-digit typing accuracy rate of four computational tools on different sequence depth.

According to Figure 2, the accuracy of HLA typing for WES by OptiType remained at about 98.9% under all average depths. The accuracy of HLA typing for WES by Phlat increased approximately from 95.56% under >100x to 98.04% under >200x and > 300x. The accuracy of Polysolver increased from 93.7% under >100x to 94.62% under >200x, and decreased to 92.16% under >300x. Although the capture regions were similar, these changes may due to the nonuniform coverages of the HLA gene regions caused by the randomness in sequencing procedures such as DNA fragmentation and amplification.

Then, we evaluated the average depth of HLA gene regions on the accuracy of HLA typing (figure 2). The HLA typing accuracy reaches 100% from 20x for OptiType, from 100x for Polysolver, and from 200x for Phlat. Besides, the typing accuracy of Phlat and Polysolver fluctuated a lot. As a result, the average depth of HLA gene regions >20x might be regarded as a quality control index for OptiType HLA typing. The average depth of the whole exon and HLA-Ia gene regions were shown in the Supplement Table S2.

### Comparison of sample types

**Figure 3:**
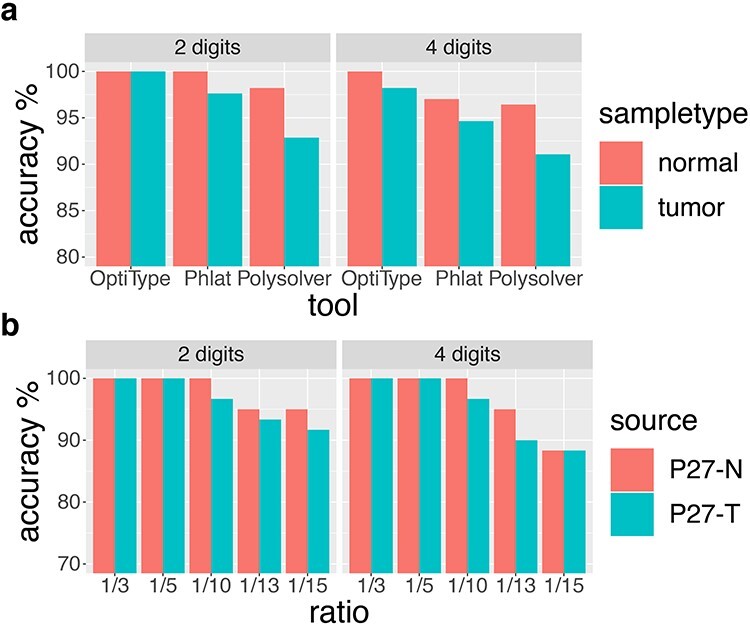
Performance comparisons in different sample types and after in silico simulation. (a) Four computational tools on WES data from control samples and tumor tissues at 2-digit and 4-digit resolution. (b). HLA typing accuracy of OptiType on WES data from control samples and tumor tissues at 2-digit and 4-digit resolution after in silico simulation.

HLA typing accuracy of WES data from tumor tissues and control PBMC samples were displayed in Figure 3a, showing the accuracy results from normal PBMC samples were mostly higher than tumor tissues. For HLA typing with OptiType, the accuracy by using normal sample and tumor tissue were all about 100% at 2-digit resolution prediction, and the accuracy were 100% for normal sample and 98.21% for tumor tissue at 4-digit resolution prediction. For Phlat, the accuracy was 100% for normal sample and 97.62% for tumor tissue at 2-digit resolution prediction, and the accuracy were 97.02% for normal sample and 94.64% for tumor tissue at 4-digit resolution prediction. For Polysolver, the accuracy was 98.21% for normal sample and 92.86% for tumor tissue at 2-digit resolution prediction, and the accuracy were 96.43% for normal sample and 91.07% for tumor tissue at 4-digit resolution prediction. These findings may recommend that it should be better to use normal samples for HLA typing.

### *In silico* simulation

Since there are fewer randomly selected reads, HLA typing errors appeared in simulated tumor samples and control samples (Figure 3b & Supplementary Table S4) and the accuracy of HLA typing by OptiType decreased from reads ratio of 1/10 to reads ratio of 1/15 at 2-digit and 4-digit resolution prediction. This might be owing to the missing signals of the undetected HLA alleles and interfering noises with the signals of detected HLA alleles. Although typing errors occurred, the accuracies of HLA typing from normal samples were still higher than tumor tissues.

## Discussion and conclusion

In this study, we have chosen four tools with good performance for HLA typing [19], and identified the potentially optimal HLA typing method tools and the probably suitable sequencing data for HLA typing methods.

For both WES data and RNA-seq data, OptiType showed the highest accuracy for HLA-Ia typing than Phlat and Polysolver with 2-digit and 4-digit resolution. In addition, HLA typing accuracy from WES data was higher than from RNA-seq data (99.11% for WES data versus 96.42% for RNA-seq data). Our results were not very consistent with Bauer’s study [19], showing 99% accuracy of HLA typing with OptiType from RNA-seq data at 4-digit resolution. This might because Bauer’s study used RNA-seq data from lymphoblastoid cell lines RNA-seq data for HLA typing, instead of tumor tissue or blood samples. Furthermore, we removed two samples with very low HLA gene expressions of HLA genes, which may reduce the accuracy of HLA typing. Then, the accuracy of OptiType for 4-digit HLA typing from RNA-seq data increased from 96.42% to 98.72%.

The average coverage of whole exome did not affect OptiType HLA typing performance. However, OptiType shows the best performance for high coverages in the HLA gene regions but that the findings were otherwise similar to Bauer’s study [19] regardless of HLA gene regions coverages. Besides, the accuracy of 2-digit and 4-digit HLA-Ia typing from control samples was higher than from tumor tissues. This may be explained by somatic mutations of the HLA genes in the tumor sample [9]. Through *in silico* simulation, we also discovered that with the reduction of reads in the HLA gene regions may be associated with increasing OptiType typing errors. This might be owing to the missing signals of the undetected HLA alleles and interfering noises with the signals of detected HLA alleles.

This study involved with several limitations. Firstly, there were still a few patients had distinct HLA-Ia typing results in tumor or control samples by OptiType in our other studies. We utilized Polysolver as a secondary analysis for these inconsistent samples and developed a scoring algorithm to integrate the results by OptiType and Polysolver to obtain the most reliable HLA typing. We will verify the efficacy of the integrated scoring program in the future. The code and manual are available on this website (https://github.com/YCBIO/hla_typing_integration_tools). Secondly, this study only discussed the 4-digit resolution prediction for HLA-Ia genes. As high-resolution HLA typing are necessary for describing neoantigens to differentiate the patients who can benefit from immunotherapy, we will continue with the further development of higher resolution typing tools such as 6-digit and 8-digit resolution. Additionally, HLA-II genes have been shown to be involved with the efficacy of immunotherapy strategies [7]. But current tools for type II HLA typing were of low accuracy [6]. We plan to work on HLA-II typing tools by evaluating the performance of existing HLA-II typing tools or develop new HLA-II typing tools in the future.

In conclusion, OptiType by using WES techniques from control samples can give probably superior performance for HLA-Ia typing. We expect that more accurate and fast prediction methods for both HLA-I and HLA-II genes at higher resolution in the future.

## Materials and Methods

### Whole exome sequencing and RNA sequencing

Whole exome sequencing for the FFPE tumor tissue and matched normal samples and RNA sequencing for tumor samples were carried out from 28 colorectal cancer or melanoma patients. PBMC from peripheral blood was served as normal sample. Genomic DNAs were from tumor tissue and blood was respectively extracted using the Qiagen DNA FFPE and Qiagen DNA blood mini kit (Qiagen). RNA-seq was extracted from FFPE tumor tissue slides using RNeasy FFPE Kit (Qiagen) (Supplementary Table S1).

WES sequencing libraries were constructed using Illumina Nextera Rapid Capture Exome kit (Illumina Genetic Ltd., USA) and procedures were performed on an Illumina HiSeq4000 platform with 150 bp paired-end reads at the average depth of 150X coverage. Total RNA sequencing libraries were constructed using Illumina TruSeq RNA Access kit (Illumina, USA) and sequencing procedures were performed on an Illumina HiSeq4000 platform with 150 bp paired-end reads at the average depth of 75 million reads.

### Gold standard

To compare performances of the 4 selected HLA typing computational programs, HLA genotyping data at 4-digit resolution using PCR-SBT method with SMRT technology was obtained from each patient and was used as the golden standard. Class Ia (HLA-A, B, C) genes were amplified by Polymerase Chain Reaction (PCR) with specific primers using genomic DNA as a template. QIAamp 96 DNA Blood Kit (QIAGEN) is used according to the manufacturer’s protocol to purify high-molecular-weight DNA. All steps of DNA extraction are processed using JANUS or other liquid handlers. Sequencing templates (DNA libraries) for PacBio are prepared according to the manufacturer’s protocols.

### Quality Control and Reads Mapping

Quality control was performed on the raw sequencing data using Fastp [20] with default parameters. Fastp will automatically trim adapter sequences which detected by finding the overlap of each pair. Low-quality bases were trimmed when the average is less than 20. PolyG in the read tails were detected and trimmed. The WES trimmed reads were aligned to a reference genome (NCBI human genome assembly hg19 and HLA reference) using the Burrows-Wheeler Aligner (BWA) program [21]. Each alignment was assigned with a mapping quality score by BWA which generated a Phred-scaled probability that the alignment is correct.

### Patch optimization

When running OptiType with too many reads, a memory error saying”/home/osboxes/seqan/include/seqan/basic/basic_exception.h:363 FAILED!” frequently occurred and was mentioned on GitHub (https://github.com/FRED-2/OptiType/issues/71).

To tackle this problem, we designed a patch for OptiType program. After initially extract the reads that can be aligned to the HLA sequence, OptiType with our patch can perform HLA typing successfully. The source code and User manual were shown online (https://github.com/YCBIO/optitype_patch).

### Performance assessment

In this study, all 4 tools were running at default settings. Gene regions of HLA-A (chr6:29910246-29,913,661), HLA-B (chr6:31321648-31,324,989), HLA-C (chr6:31236525-31,239,913) were used to calculate the average depth. The average depth of total exon and HLA gene regions was count using locally developed Python program.

According to a previous study [19], accuracy is calculated over all the samples an all the alleles as the following formula:

$$\mathrm{Accuracy}=\frac{\#\mathrm{RightAlleles}}{\#\mathrm{RightAlleles}+\#\mathrm{WrongAlleles}+\#\mathrm{NAAlleles}} $$

‘Right Allele’ related to those HLA alleles called by computational tools consistent with PCR-SBT determined allele. ‘Wrong Allele’ means those HLA alleles called by computational tools inconsistent with PCR-SBT determined allele. ‘NA Allele’ referred to those HLA alleles which cannot be detected by NGS with computational tools.

### Gradient data simulation

We selected the WES data of tumor tissues and paired normal samples (P27-T, P27-N) from one patient to do *in silico* simulation to perform HLA typing by using OptiType. The sequencing depth of HLA gene regions of both tumor tissues and paired normal samples were similar of >100x (Supplementary Table S2). Reads aligned to the HLA gene regions were randomly chosen at gradient ratios of 1/3, 1/5, 1/10, 1/13 and 1/15 with 10 replicates for each ratio.

**Key Points**

- This article conducted the performance evaluations of four widely used HLA typing tools (OptiType, Phlat, Polysolver and seq2hla) by predicting HLA class Ia genes from WES and RNA-seq data of 28 cancer patients.
- For both WES data and RNA-seq data, OptiType showed the highest accuracy for HLA-Ia typing than the other three computational tools with 2- and 4-digit resolution.
- HLA typing accuracy from WES data higher than from RNA-seq data (99.11% for WES data versus 96.42% for RNA-seq data). The accuracy of HLA-Ia typing by OptiType can reach 100% with the average sequencing depth of HLA gene regions >20×. Besides, the accuracy of 2- and 4-digit HLA-Ia typing based on control samples was higher than tumor tissues.
- After *in silico* simulation, the accuracy of HLA typing decreased along with the reduction in reads number in HLA-Ia gene regions at 2- and 4-digit resolution prediction.

## Supplementary Material

Supplementary_Table_bbaa143

## Abbreviations

HLA, human leukocyte antigen; HLA-I, HLA class I genes, including HLA-Ia and HLA-Ib; HLA-Ia, HLA-A, HLA-B and HLA-C; HLA-Ib, HLA-E, HLA-F and HLA-G; HLA-II, HLA class II genes, including HLA-DP, HLA-DQ and HLA-DR; MHC, human major histocompatibility complex; LOH, loss of heterozygosity; WGS, whole-genome sequencing; WES, whole-exome sequencing; PBMC, peripheral blood mononuclear cells; RNA-seq, RNA sequencing; PCR, polymerase chain reaction; PCR-SSP, polymerase chain reaction-sequence specific primer; PCR-SSO, polymerase chain reaction-sequence specific oligonucleotide probes; PCR-SBT, polymerase chain reaction-sequencing based typing; SMRT, single-molecule real-time sequencing; FFPE, formalin-fixed, paraffin-embedded.

## Notes

**Xiaofan Su**, PhD, Director of Yucebio Cancer Translational Research Institute and Chief Medical Officer for Yucebio Technology Co., Ltd., has been worked as Medical Director for Johnson & Johnson Co., Ltd., AstraZeneca INC., Kingmed Diagnostics Co., Ltd.

**Jian Yi**, a member of Cancer Translational Research Institute, YuceBio Technology Co., Ltd., Shenzhen, China.

**Longyun Chen**, a member of Cancer Translational Research Institute, YuceBio Technology Co., Ltd., Shenzhen, China.

**Yajie Xiao**, a member of Cancer Translational Research Institute, YuceBio Technology Co., Ltd., Shenzhen, China.

**Zhikun Zhao**, a member of Cancer Translational Research Institute, YuceBio Technology Co., Ltd., Shenzhen, China.

## References

[1] Hicklin DJ , Marincola FM, Ferrone S. HLA class I antigen downregulation in human cancers: T-cell immunotherapy revives an old story. Mol Med Today 1999. 10.1016/s1357-4310(99)01451-3 10203751

[2] Wright P , Nimgaonkar VL, Donaldson PT, et al. Schizophrenia and HLA: a review. Schizophr Res 2001. 10.1016/s0920-9964(00)00022-0 11163540

[3] Toni Ho G-G , Heinen F, Stieglitz F, et al. Dynamic interaction between immune escape mechanism and HLA-Ib regulation. Immunogenetics 2019.

[4] Matey-Hernandez ML , Brunak S, Izarzugaza JMG. Benchmarking the HLA typing performance of Polysolver and Optitype in 50 Danish parental trios. BMC Bioinformatics 2018;19:1–12. 29940840 10.1186/s12859-018-2239-6 PMC6019707

[5] Szolek A , Schubert B, Mohr C, et al. OptiType: precision HLA typing from next-generation sequencing data. Bioinformatics 2014;30:3310–6. 25143287 10.1093/bioinformatics/btu548 PMC4441069

[6] Schumacher TN , Schreiber RD. Neoantigens in cancer immunotherapy. Science (80- ) 2015;348:69–74. 10.1126/science.aaa4971 25838375

[7] Perea F , Bernal M, Sánchez-Palencia A, et al. The absence of HLA class I expression in non-small cell lung cancer correlates with the tumor tissue structure and the pattern of T cell infiltration. Int J Cancer 2017. 10.1002/ijc.30489 27785783

[8] Garon E , Requena D, Chowell D, et al. Patient HLA class I genotype influences cancer response to checkpoint blockade immunotherapy. Science (80- ) 2017;359:582–7. 10.1126/science.aao4572 PMC6057471 29217585

[9] Shukla SA , Rooney MS, Rajasagi M, et al. Comprehensive analysis of cancer-associated somatic mutations in class i HLA genes. Nat Biotechnol 2015;33:1152–8. 26372948 10.1038/nbt.3344 PMC4747795

[10] Althaf MM , El Kossi M, Jin JK, et al. Human leukocyte antigen typing and crossmatch: a comprehensive review. World J Transplant 2017. 10.5500/wjt.v7.i6.339 PMC5743871 29312863

[11] Dunn PPJ . Novel approaches and technologies in molecular HLA typing. Methods Mol Biol 2015. 10.1007/978-1-4939-2690-9_18 26024638

[12] De Santis D , Dinauer D, Duke J, et al. 16th IHIW : review of HLA typing by NGS. Int J Immunogenet 2013. 10.1111/iji.12024 PMC4793271 23302098

[13] Boegel S , Löwer M, Schäfer M, et al. HLA typing from RNA-Seq sequence reads. Genome Med 2012;4. 10.1186/gm403 PMC4064318 23259685

[14] Robinson J , Barker DJ, Georgiou X, et al. IPD-IMGT/HLA database. Nucleic Acids Res 2020. 10.1093/nar/gkz950 PMC7145640 31667505

[15] Bai Y , Wang D, Fury W. PHLAT: inference of high-resolution HLA types from RNA and whole exome sequencing. Methods Mol Biol 2018;1802:193–201. 29858810 10.1007/978-1-4939-8546-3_13

[16] Langmead, Salzberg SL. Bowtie2. Nat Methods 2013.

[17] Weese D , Holtgrewe M, Reinert K. RazerS 3: faster, fully sensitive read mapping. Bioinformatics 2012. 10.1093/bioinformatics/bts505 22923295

[18] Robinson J , Soormally AR, Hayhurst JD, et al. The IPD-IMGT/HLA database - new developments in reporting HLA variation. Hum Immunol 2016. 10.1016/j.humimm.2016.01.020 26826444

[19] Bauer DC , Zadoorian A, Wilson LOW, et al. Evaluation of computational programs to predict HLA genotypes from genomic sequencing data. Brief Bioinform 2018;19:179–87. 27802932 10.1093/bib/bbw097 PMC6019030

[20] Chen S , Zhou Y, Chen Y, et al. Fastp: an ultra-fast all-in-one FASTQ preprocessor. Bioinformatics 2018. 10.1093/bioinformatics/bty560 PMC6129281 30423086

[21] Li H , Durbin R. Fast and accurate long-read alignment with burrows-wheeler transform. Bioinformatics 2010. 10.1093/bioinformatics/btp698 PMC2828108 20080505

